# Effectiveness of tailored multichannel interventions on weight loss among adolescents: a randomized controlled trial

**DOI:** 10.3389/fpubh.2026.1733027

**Published:** 2026-03-27

**Authors:** Mahmoud Ahmed Elsheikh, Mohamed Ali Zoromba, Tarek Selim, Mohamed Hussein Ramdan Atta, Narges Fathi Mohamed, Samar Othman Abdelazim, Heba Emad El-Gazar, Osama Albasheer, Ali Daif Abousoliman, Lamiaa Saad Abdallah

**Affiliations:** 1 Nursing Department, College of Applied Medical Sciences, Prince Sattam bin Abdulaziz University, Wadi Addawasir, Riyadh, Saudi Arabia; 2 College of Nursing, Prince Sattam bin Abdulaziz University, Al Kharj, Riyadh, Saudi Arabia; 3 Community Health Nursing, Faculty of Nursing, Mansoura University, Mansoura, Dakahlia, Egypt; 4 Faculty of Nursing, Mansoura National University, Faculty of Nursing, Mansoura National University, Dakahlia, Egypt; 5 Graduate School of Biomedical and Health Sciences, Hiroshima University, Hiroshima, Japan; 6 Faculty of Nursing, the British University in Egypt, Cairo, Egypt; 7 College of Nursing, Imam Mohammad Ibn Saud Islamic University (IMSIU), Riyadh, Saudi Arabia; 8 Family and Community Medicine Department, College of Medicine, Jazan University, Jazan, Saudi Arabia; 9 Community Health Nursing, Faculty of Nursing, Cairo University, Cairo, Egypt

**Keywords:** adolescents, digital intervention, college-based telephone calls, health belief model, multichannel intervention, tailored, weight loss, WhatsApp text messages

## Abstract

**Background:**

Several weight loss interventions have been provided for adolescents. However, these interventions were often not adequately tailored to their perception. This study aims to investigate the effectiveness of tailored multichannel interventions on weight loss among adolescents.

**Methods:**

This study is a randomized controlled trial. A total of 279 adolescents, whose Body Mass Index (BMI) was≥25 kg/m^2^, were recruited in January 2025 from eight non-medical colleges at Mansoura University, Mansoura, Egypt. For the intervention group (IG; *n* = 133), an intervention was developed based on the Health Belief Model (HBM). The intervention has been designed and administered by a multidisciplinary team for 5 months, until June 2025, via in-person meetings, telephone calls, and digital messages. The control group (CG; *n* = 146) received basic education over two face-to-face sessions. The primary outcome is the mean difference in BMI. Secondary outcomes include self-administered HBM constructs. All outcomes were assessed at baseline (T0), 2 months (T1), and 5 months (T2).

**Results:**

The independent *t*-test revealed statistically significant differences between the groups in the scores of the BMI at T2 (−1.75, 95% CI: −2.48 to −1.02, *p* < 0.001, Cohen’s *d* = −0.57). Additionally, two-way repeated measures ANOVA demonstrated a statistically significant interaction effect (Group × Time), *F*(1.24, 342.36) = 49.72, *p* = <0.001, partial η^2^ = 0.15, as well as statistically significant differences within-group, *F*(1.24, 342.36) = 29.73, *p* = <0.001, partial η^2^ = 0.10. Regarding HBM, the intervention produced statistically significant main effects between and within groups over time for perceived susceptibility, benefits, self-efficacy, and internal cues to action (all *p* < 0.05).

**Conclusion:**

Adolescents in the IG experienced significant weight loss and a positive change in their perception of weight management. The study findings suggest that such tailored multichannel interventions be integrated with other community-based participatory approaches to enhance external cues to action and overcome the perceived barriers in the long term.

**Clinical trial registration:**

https://clinicaltrials.gov/study/NCT06767072, NCT06767072.

## 1 Introduction

Adolescent overweight is a global public health issue with rapidly increasing prevalence (1) and negative health consequences (2). Although it is preventable, rates have quadrupled worldwide since 1990 (3). This significant rise has been observed across high-income, middle-income, and low-income countries, indicating global shifts in dietary practices, physical activity, and living conditions (4). Being overweight in adolescence often leads to negative health effects in adulthood (5). It is associated with a higher risk of cardiovascular disease, psychiatric issues, type II diabetes, fatty liver, and other metabolic syndromes (6). Overweight adolescents also face emotional disorders (7), social barriers, and economic burdens (8).

Adolescent overweight results from an interaction of multiple relevant factors. High intake of sugar-sweetened beverages, calorie-dense, nutrient-poor diets, inadequate physical activity, and extended screen time are major individual contributors (9). Low self-efficacy, emotional eating disorders, perceived body image, and peer influences further affect adolescents’ healthy choices and weight management-related practices (10). Broader environmental and social contexts, such as urbanization, lack of access to healthy foods, marketing of unhealthy foods, family dietary patterns, socioeconomic circumstances, and school setting environments, all of which reinforce individual-level factors and contribute to an obesogenic environment (11).

Adolescence is a critical period. Dietary habits and lifestyle behaviors are shaped, making it an ideal time for interventions that aim at promoting healthy weight management (12). Several interventions have been conducted to enhance weight management behavior among adolescents in many countries (13). However, the effectiveness of past interventions has shown inconsistent outcomes (14). Compliance with weight-loss interventions varies due to multiple factors, mostly associated with the design and implementation of the interventions (15).

Systematic reviews reveal debate regarding the best way to motivate adolescents to manage their ideal body weight (15). Empirical evidence shows that tailored interventions are often more effective than standardized interventions for reducing overweight among adolescents. Tailored interventions account for individual differences in lifestyle, preferences, perceptions about health, self-efficacy, and challenges (16). While standardized interventions can provide a solid foundation, tailoring strategies to individual needs often leads to more sustainable behavior changes and better health outcomes (17).

The literature suggests that interventions delivered through multiple channels—such as social media, face-to-face interactions, and telephone calls—are often more effective in addressing overweight among adolescents (18). Adolescents are highly engaged with digital platforms, making them an effective channel and providing daily reminders or motivation (19). Incorporating face-to-face interactions into digital platforms establishes personal connections and offers in-depth discussions (20). While telephone calls provide a direct and accessible line of communication (5).

The literature highlighted that tailored interventions could achieve positive outcomes. However, there is a lack of rigorous research incorporating weight management strategies within the scope of practice (21). Ongoing support and encouragement can significantly motivate adolescents to achieve weight loss objectives (22). Assessing adolescents’ unique perceptions and challenges and designing tailored plans fosters sustainable weight loss (23). Improving research in these ways would provide a strong evidence base to expand the role of tailored strategies in the prevention and management of overweight (24).

Despite literature recommendations, the effectiveness of interventions that adopted tailored and multichannel approaches has not been adequately assessed in developed and developing countries. For instance, Egypt currently ranks among the countries with the highest rates of overweight worldwide (25). The prevalence of overweight among Egyptians is 39.82% (26). According to the updated systematic review, the prevalence of overweight among adolescents is 13.3% (27). Moreover, overweight-related health consequences among adolescents keep rising. According to a national study, adolescents’ overweight is associated with cardiometabolic risks and social and psychological negative consequences (28).

The literature concerning the effectiveness of such interventions is limited. There is a lack of knowledge regarding their usefulness across various contexts (17). Therefore, the current study suggests that a tailored, multichannel intervention is likely to have a positive effect on weight loss among adolescents in Egypt.

### 1.1 Conceptual framework of tailored multichannel intervention: applying health belief model to weight loss

**Figure 1:**
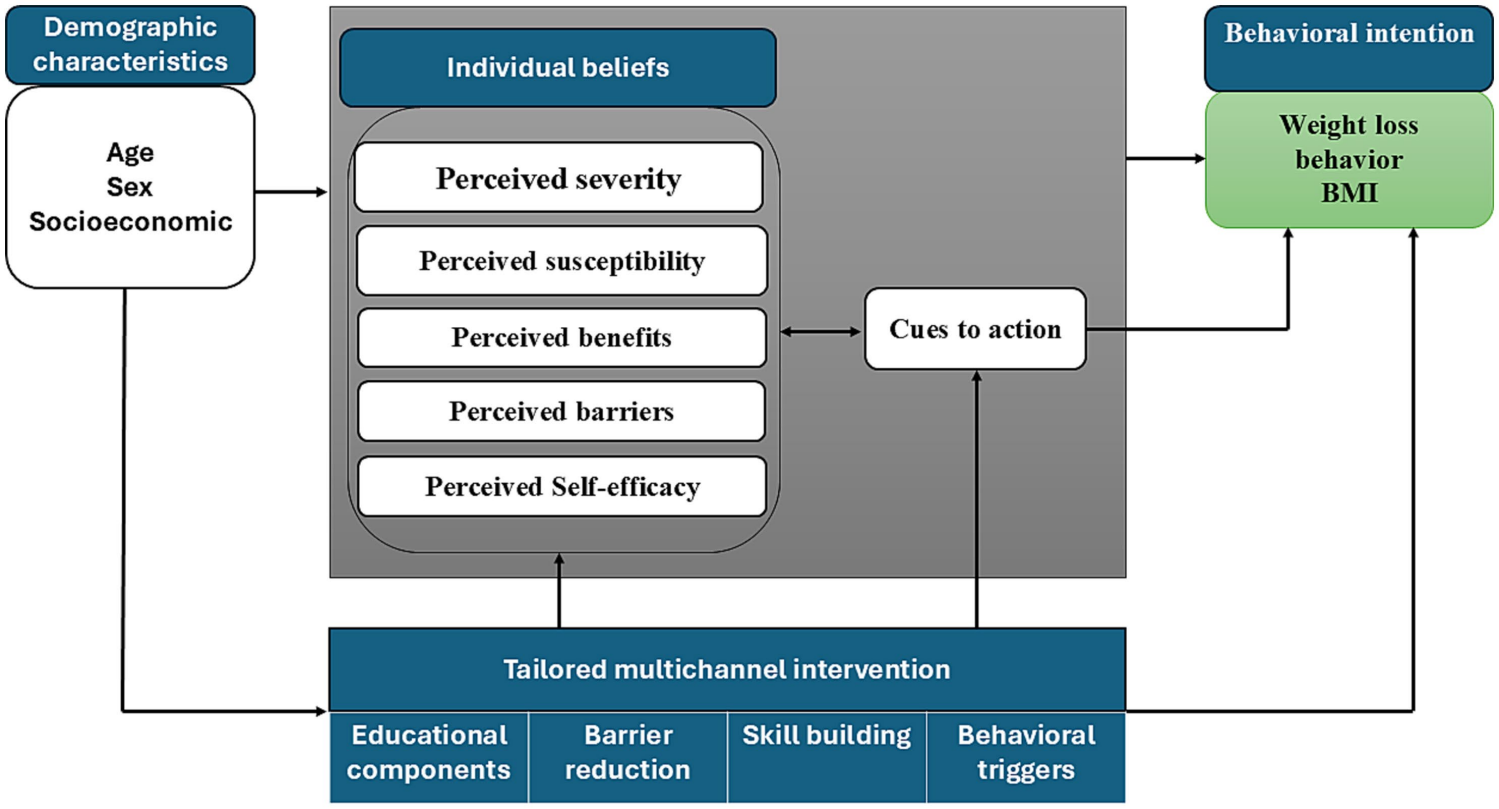
Conceptual framework.

The Health Belief Model (HBM) is a framework that analyzes and predicts health behaviors by addressing individual beliefs (29). HBM has been successfully adapted to topics across various disciplines (30, 31). For weight loss interventions, HBM constructs can provide a structured approach to understanding how individuals perceive weight loss (32) and what intervention motivates them to adopt healthier behaviors (33). The conceptual framework of the current study (Figure 1) outlines the relationship between HBM constructs and weight loss among adolescents, as well as how the proposed intervention influences the adolescents’ perception and beliefs to motivate them to adopt weight loss behaviors. The fundamental constructs of the HBM are perceived severity, perceived susceptibility, perceived benefits, perceived barriers, perceived self-efficacy, and cues to action (29). Prior research showed that the aforementioned variables directly affect the behavioral intention of weight management among adolescents (34) and are crucial to the effectiveness of weight-loss programs (35).

A systematic review reported that although many behavioral weight loss interventions demonstrated evidence-based strategies, only a few studies employed a theoretical base (36). Based on HBM, the current study hypothesizes that fostering positive health beliefs regarding a healthy weight through education and training positively influences weight management behavior (37). Considering individual characteristics of adolescents and their perceptions, the intervention addresses tailored intervention strategies. These strategies include providing educational information, reducing barriers, skill-building, and addressing behavioral triggers. The components of the intervention are explained clearly in Table 1.

**Table 1:** The framework of the intervention contents.

In-person, Face-to-face college meeting (60 min)	Telephone call (20–30 min)	WhatsApp interactive message (15–20 min)
Objectives (Educational information): Provide basic information on balanced nutrition. Discuss the individual nutritional requirements and risks of being overweight.	Objectives (Skill building and barrier reduction): Mitigate perceived obstacles to weight management. Build weight management skills and enhance self-efficacy to sustain weight loss.	Objectives (Behavioral triggers): Initiate regular behavioral triggers. Remember monthly tips to maintain healthy behavior.
Severity of being overweight Susceptibility of being overweight Benefits of adopting weight loss behavior	Perceived barriers to adopting weight loss behavior Solutions to overcome barriers	Remember your weight loss goal should be time limited (time frame). Self-monitoring: Weekly Calculation of BMI and interpreting results (manual and using BMI calculators).
How many calories do you need using the BMR calculator Acceptable Macronutrient Distribution Ranges (AMDR) Essential Vitamins Water and Minerals	Change your eating habits; be more aware of how much you eat and choose affordable diet/meal options. Preparing low-calorie, high-nutrition meals. Personalized beginner-level exercise plans; adopting affordable exercise options	Remember: The types of foods eaten regularly are related to whether weight gain or loss occurs over time. Remember: regular physical activity alone is not likely to lead to weight loss, but it has numerous health benefits. Review the diet and physical activity personalized plan
Revise your created diet and exercise plan.	Reading food labels and making healthier choices. Using rewards for achieving goals	Identify what causes you to feel the need to eat. Rewarding proper eating behavior can help in developing better habits. The aim is to reward yourself for replacing undesirable habits with positive ones, not for losing weight.
The current behavior and the progress toward behavioral goals. Review the successful strategies used and challenges experienced.	Be aware of family and peer influence: getting support for healthy behavior change Discuss challenges and improvement strategies.	Remember: the types of foods associated with weight gain. Remember: the types of foods associated with maintaining a healthy weight.
Enforcement of strategies found beneficial Where to get reliable and valid information	Substitute negative thoughts with positive thinking. Avoid temptations, handle slips, and stay motivated Reduce stress Organized and adequate sleep/rest	Drink an adequate amount of water. Unnecessary dietary supplements are not recommended Include exercise in everyday schedules. Track the activity and food log.

In brief, educational information aims to enhance the perception of adolescents about the health risks of being overweight (severity and susceptibility) and the benefits of weight loss. Perceived severity indicates an adolescent’s belief about the seriousness of the negative health effects of being overweight, ranging from illness to death (38). The current intervention aims to emphasize the severe health outcomes of being overweight (e.g., chronic diseases and reduced life expectancy) and the impact on quality of life (e.g., physical limitations and social stigma) (39). This can enhance perceived severity and create greater behavioral intention of weight management (40). Perceived susceptibility refers to adolescents’ beliefs about the probability of developing health problems due to being overweight (32). The intervention proposes that adolescents who perceive themselves as being at high risk of overweight-related complications may be more motivated to lose weight and more likely to engage in preventive measures (10).

Barrier reduction as a component of the current intervention aims to help adolescents identify and mitigate perceived barriers. Perceived barriers to a healthy lifestyle significantly influence the behavioral intentions to weight management (41) and often hinder adherence to healthy behaviors (42). Addressing these barriers with solutions enhances adherence to healthy nutrition and active exercise (43).

The intervention focuses on weight management skill-building, aiming to improve adolescents’ self-efficacy, which may be observed in their ability to lose weight successfully and maintain healthy behaviors (44). Self-efficacy in the scope of weight management refers to an individual’s belief regarding their ability to comply with a balanced diet and perform regular physical activities, even under challenging conditions (45). The current study suggests that building weight management skills increases adolescents’ self-efficacy, leading to higher engagement in consistent behavior (40).

The intervention also includes behavioral triggers, incorporating cues to action. It is proposed that behavioral triggers will motivate the perception of external or internal cues to action. Subsequently, this will prompt an adolescent to initiate and maintain weight loss behaviors (10). The current study suggests a direct effect of cues to action on engagement in health behavior. Additionally, cues to action mediate the effects of perceived severity, susceptibility, benefits, barriers, and self-efficacy on weight management behavior (43).

### 1.2 Aim and hypothesis

The proposed study aimed to evaluate the effectiveness of tailored multichannel interventions on weight loss among adolescents. The primary hypothesis is that adolescents who receive a tailored multichannel intervention (intervention group) will experience a reduced BMI compared to those who receive basic education on weight management (control group).

#### 1.2.1 Secondary objective

The proposed study assesses the change in adolescents’ perception regarding weight management and loss, in terms of perceived severity, perceived susceptibility, perceived benefits, perceived barriers, perceived self-efficacy, and cues to action.

## 2 Methods

### 2.1 Trial design

The study design is a prospective, randomized controlled trial (RCT). The study was registered at ClinicalTrials.gov (number NCT06767072).

### 2.2 Setting and participants

The recruitment setting for this study is eight non-medical colleges at Mansoura University (commerce, education, arts, rights, engineering, computer and information, fine arts, and special education). Mansoura University is located in Mansoura City, the capital of Dakahlia, Egypt. Students in the first and second grades of college who were 17–19 years old and had a BMI equal to 25 or higher at the baseline assessment were eligible to participate in the study. Students were excluded from the study if they had one of the following criteria. (1) Students who disclose a physical condition, chronic disease, or handicap that hinders their ability to engage in physical activity or reduce extra body weight, (2) Students with a history of mental health disorders, (3) Students taking medication that causes weight gain, or (4) Students who participate in another weight loss program.

### 2.3 Recruitment

Participants were approached by teachers at the selected eight colleges in January 2025. Teachers were contacted, and the aim of the study was explained. The teachers announced the study to the students. Second, the researchers visited the colleges and explained the purpose of the study to students. Students who agreed to participate were asked to provide written informed consent. Participants’ weight and height were measured, and BMIs were calculated. Eligible participants with a BMI of 25 or higher were enrolled in the study.

### 2.4 Randomization and allocation

A randomized method has been used to allocate participants to the IG or the CG. Firstly, the study was announced at the selected schools. Within each selected college, students who expressed interest in participation were screened for the inclusion criteria. Eligible participants were randomly assigned to IG or CG by an independent researcher using computer-generated random numbers. The study employs an open-label design without concealment, given the nature of the study. Regarding blinding, neither care providers nor outcome assessors were blinded.

### 2.5 Intervention tailoring and fidelity

The intervention was formulated by researchers from various fields, including specialists in community health, psychiatry, and nutrition (interdisciplinary team). The designing procedure for each individual involved the following consecutive steps: (1) the nurses asked the participants to complete HBM constructs, which are used to collect data on each unique perception regarding overweight and weight loss (10); (2) data of adolescent health beliefs are summarized by the interdisciplinary team.; (3) the interdisciplinary team then creates an intervention plan; (4) the intervention plan, outlined by realistic goals and applicable strategies, and shared with the participant. The completed Template for Intervention Description and Replication checklist (TIDieR) checklist has been completed and provided as Supplementary file 1.

The intervention modules are fundamentally consistent with the contents of the nutritional behavior program published elsewhere (46, 47), which corresponds with the guidelines of the National Nutrition Institute. The current intervention also takes into account evidence-based practice and expert recommendations for treating adolescents’ overweight (48–50). It considers the guidelines of successful weight loss (51) and guidelines of the American Heart Association (AHA) (52).

Table 1 shows a summary of the content framework and the schedule of intervention delivery. Ten nurses administered the proposed intervention over a five-month period, comprising five face-to-face meetings, five phone calls, and five WhatsApp text messages. Each month included a 60-min college educational session, a 20–30 min telephone counseling call focused on skills building and barrier reduction, and a 15–20 min WhatsApp interactive message serving as a behavioral trigger. The intervention sessions were administered at a 10-day interval. The intervention sessions were strategically planned to build knowledge, enhance self-efficacy, reduce perceived barriers, and sustain motivation for long-term behavior change.

First month: introduction to weight-related health beliefs and self-monitoring.

The first month focused on foundational health beliefs by addressing the severity and susceptibility associated with being overweight, as well as the perceived benefits and barriers to adopting weight loss behaviors. Strategies were introduced to participants to overcome these barriers and engage in weekly self-monitoring practices, including the calculation and interpretation of BMI using both manual and digital tools. This month aims to raise awareness and motivation for behavior change.

Second month: nutrition and exercise awareness.

In the second month, the focus shifted to personalized nutrition education. Adolescents learned how to calculate the number of required calories using the Basal Metabolic Rate (BMR) calculator and received guidance on Acceptable Macronutrient Distribution Ranges (AMDR). Adolescents were encouraged to follow weight loss goals, improve their eating habits, and incorporate more physical activity. Furthermore, the participants were taught practical components to develop personalized exercise plans and meal preparation strategies that emphasized low-calorie, high-nutrition choices. The importance of consistent behavior and establishing time-bound goals was highlighted.

Third month: making informed dietary decisions and using positive reinforcement.

During the third month, the participants engaged in individualized plans for a healthy diet and physical exercise. They were educated on how to interpret food labels and make healthier food choices. The behavioral focus was on identifying eating triggers and using non-food rewards to reinforce positive changes. Emphasis was placed on rewarding healthy behavior modifications rather than weight loss outcomes, promoting intrinsic motivation.

Fourth month: navigating external influences and assessing weight loss.

During this month, the nurses addressed the influence of family and peers, emphasizing the need for a supportive environment. Participants were instructed to assess the accomplishment of behavioral goals. They were advised to review the challenges they experienced and the strategies they used. Educational content helped differentiate between foods associated with weight gain and those that support a healthy weight, reinforcing the value of evidence-based dietary choices.

Fifth month: sustaining long-term behavior change.

The final month aimed to reinforce healthy lifestyle habits and build resilience against common challenges. Adolescents were taught how to seek reliable information sources, manage temptations, and adopt strategies for stress reduction, positive thinking, and sufficient rest. They were also advised to maintain hydration and avoid unnecessary dietary supplements. These components supported the transition to long-term maintenance of healthy behaviors.

### 2.6 Procedures to improve the adherence level

The nurses underwent a comprehensive training. The training employed various methodologies, such as demonstrations and redemonstrations, group discussions, role-plays, and both in-person and virtual lectures. Training emphasized data collection techniques and instrument utilization, along with the development of a tailored plan with shared objectives.

The study included several methods to improve adherence. First, the intervention provided to adolescents. It is assumed adolescents are willing to maintain healthy body weight and body image, thus a lower dropout rate is anticipated. Second, tailored interventions to the perceived beliefs may ensure compliance and raise the intervention effectiveness. Third, different modes of delivery were used in short time intervals (10 days) to improve adherence levels. Each intervention nurse was assigned to a group of 13 or 14 participants in the IG. The nurse is accountable for administering the intervention, following up with the participants, answering the participants’ questions, or providing continuous feedback. Fourth, the interdisciplinary team evaluated the progress of the intervention and offered continuous mentoring of participants’ attendance, call completion, WhatsApp engagement, risks, or harms.

There were no risks, adverse events, or negative psychological symptoms of the intervention reported.

### 2.7 Control group

The CG was provided with general information on being overweight and general advice on managing their weight. These were explained to them during two college visits. CG participants did not receive the designed multichannel intervention.

### 2.8 Outcomes and instruments

#### 2.8.1 Primary outcome: anthropometric measurement

The primary outcome is the between-group difference in change in body mass index, reflecting the clinical objective of assessing intervention-attributable weight change. This approach is commonly used in randomized trials and offers a direct and interpretable measure of effectiveness.

Weight and height were assessed using a numerical electronic scale and a wall-mounted tape measure, respectively. BMI was calculated [BMI = weight (in kg)/height (in m)^2^] and interpreted based on WHO guidelines and using BMI charts (53). Overweight is considered when a BMI is 25 kg/m^2^ or over (10, 54).

#### 2.8.2 Secondary outcome: health belief model construct

The key constructs of the HBM include 89 statements (55). Thirteen questions assessed perceived severity, 7 questions assessed perceived susceptibility, 14 questions addressed perceived barriers, 13 questions on perceived benefits, 12 questions on cues to action, 18 questions evaluated self-efficacy in dieting, 7 questions on self-efficacy in exercise, and 5 questions on the behavioral intention of weight management (dieting and exercising). Between-group differences in change in these constructs are the secondary outcomes of the study. All items are self-reported and scored on a 5-point Likert scale, with 1 representing “strongly disagree” and 5 indicating “strongly agree.” Higher scores denoted a more positive belief toward overweight preventive behaviors. The tool was translated into Arabic and adapted to the culture. Based on Cronbach’s alpha, the overall reliability of the tool was 0.92 (56). The current study demonstrates high overall reliability of the tool, with a Cronbach’s alpha of 0.93. Each construct and its subscale also show strong reliability (0.84–0.94).

### 2.9 Data collection

All data related to primary and secondary outcomes were collected at baseline (T0), at 2 months (T1), and at 5 months (T2) after the start of the intervention. BMI was calculated and documented by the nurses at three time-points. Also, participants were required to complete the HBM construct.

### 2.10 Ethical considerations

Institutional Review Board approval was obtained from the Faculty of Nursing at Mansoura University (IRB 0689/30/12/2024). The study adhered to ethical principles outlined in the Declaration of Helsinki, protecting participants’ rights and wellbeing. All participants were provided with oral and written details on the study before being enrolled. The study participants were informed that all data collected is for research purposes only and that they have the right to withdraw from the study at any time. Informed written consents had been obtained from the enrolled participants. Data collection is fully anonymized and only accessed by authorized study staff.

### 2.11 Sample size

Using G Power software V.3.1.9.4 (Psychonomic Society, Madison, Wisconsin, United States) (57), the sample size was estimated. Assuming a difference between two independent means (two groups), a statistical power of 0.80, a confidence level of 0.95, and an effect size of 0.386, which was based on a similar previous study on weight loss using BMI (58), the sample size was 214 participants. An additional 30 participants were recruited to compensate for an estimated dropout rate of 30% (59). The total sample size was 279 participants.

### 2.12 Statistical analyses

Intention-to-treat analysis was applied in this study (60). All statistical analyses were conducted using Statistical Package for Social Sciences (SPSS) V.29.0 (IBM). Statistical significance was set at *p* < 0.05. The baseline characteristics of the two groups were compared using the *t*-test, Pearson’s χ^2^ test, or Fisher’s exact test, according to the variable type. The baseline characteristics include participants’ demographic data, such as age, sex, marital status, academic semester, residence, and family income. It also includes questions related to obesity among family members and experience in weight loss through behavior change, diet therapy, physical exercises, or medical treatment.

Independent *t*-tests were conducted to measure the efficacy of the intervention by comparing the means of the outcomes (BMI and HBM subscales) between the two groups at separate time points: T0, T1, and T2. In addition, to evaluate changes in outcomes within the groups over time (T0–T2), a two-way repeated measures analysis of variance (ANOVA) was conducted. The assumptions of the applied statistical tests were fulfilled (i.e., normality, homogeneity, and sphericity of variance) (61). Occasionally, the assumption of normality and sphericity was violated, so the Greenhouse–Geisser correction was applied (62). The last observation carried forward method was used to compensate for missing data. LOCF was specified as a conservative method consistent with an intention-to-treat analysis, proposing no future progress following dropout and thereby reducing the bias of overestimating intervention effects. Significantly, attrition was low and similar between groups across time points, reducing the differential attrition bias.

Sex and age-stratified analyses were conducted. In addition, analysis of covariance (ANCOVA) was performed to investigate the effect of the intervention on the primary outcome after adjusting for confounding variables such as age and sex of the participants.

Bias in evaluating the effects of the intervention is expected because of the study’s open-label design. A researcher who did not participate in the intervention or allocation of participants carried out the statistical analysis.

## 3 Results

**Figure 2:**
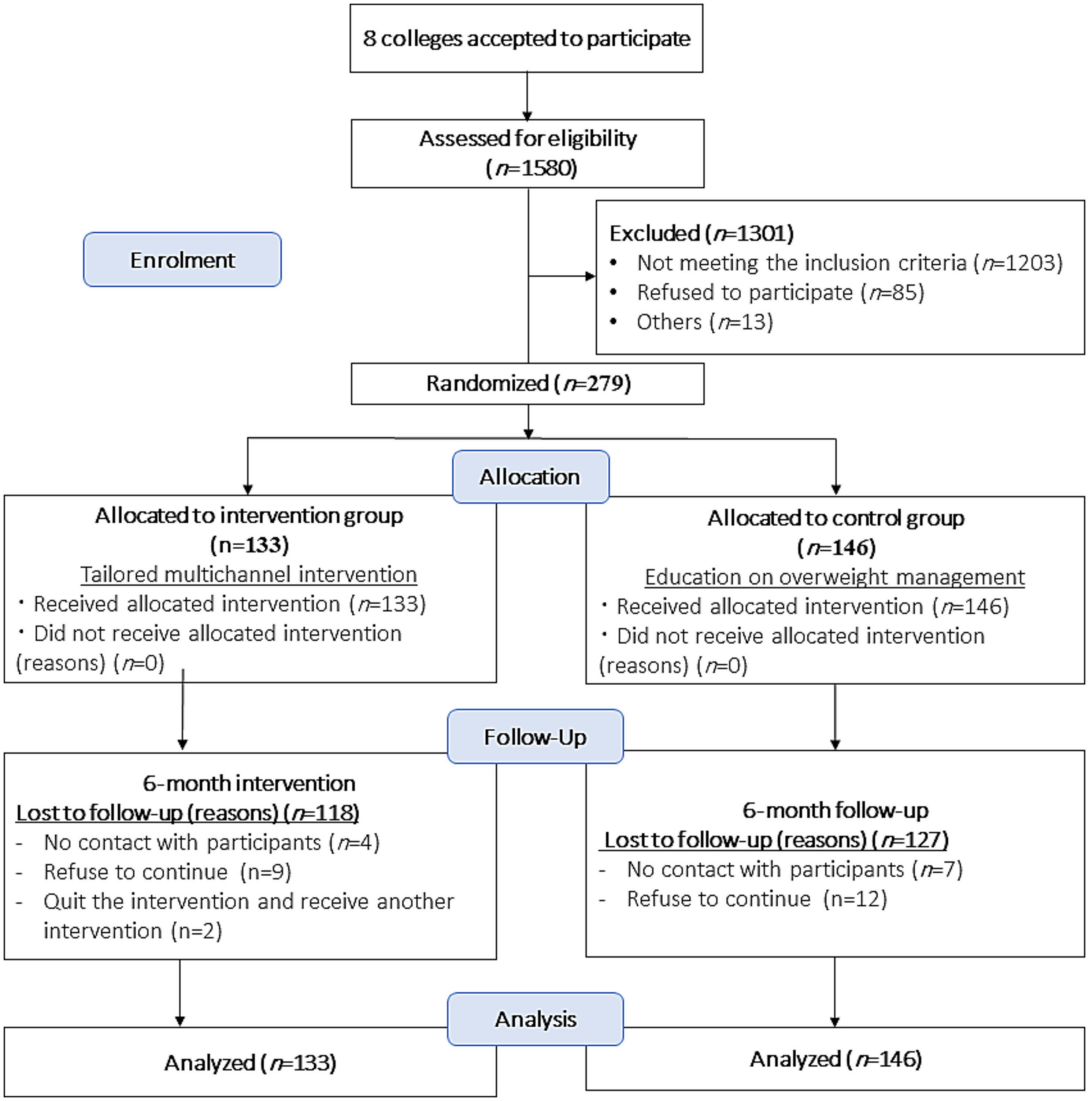
Diagram according to consolidated standards of reporting trials (CONSORT).

Figure 2 shows the CONSORT (Consolidated Standards of Reporting Trials) diagram of the study. During January 2025, a total of 1,580 participants were assessed to verify their eligibility for the study from eight colleges (education: 105, arts: 183, engineering: 170, fine arts: 254, commerce: 266, rights: 220, computer and information: 100, and specific education: 282). Of them, 279 participants from eight colleges were enrolled in the study and allocated to either the IG (*n* = 133) or the CG (*n* = 146), as illustrated in Table 2. A total of 245 (118 in the IG and 127 in the CG) completed the study within 5 months by June 2025. The data of all 279 participants were analyzed. The reasons for dropout are displayed in Figure 2, where all dropouts within the two groups occur early, before the first assessment (T1). Supplementary file 2 shows the CONSORT checklist of reporting results.

**Table 2:** Baseline assessment of adolescents (IG; *n* = 133) and (CG; *n* = 146).

Variables	Total (*n* = 279)	IG (*n* = 133)	CG (*n* = 146)	Test value	*p*-value
Demographic					
Age	17.88 ± 0.78	17.9474 ± 0.77	17.8151 ± 0.78787	1.415	0.158
Sex				0.001	0.979
Male	124 (44.4%)	59 (44.4%)	65 (44.5%)		
Female	155 (55.6%)	74 (55.6%)	81 (55.5%)		
Marital status				1.631	0.274
Single	265 (95.0%)	124 (93.2%)	141 (96.6%)		
Married	14 (5.0%)	9 (6.8%)	5 (3.4%)		
Income	7530.46 ± 3478.34	7345.86 ± 3531.34	7698.63 ± 3432.84	−0.846	0.398
Academic year				0.029	0.864
First year	163 (58.4%)	77 (57.9%)	86 (58.9%)		
Second year	116 (41.6%)	56 (42.1%)	60 (41.1%)		
College					
Fine arts	38 (13.6%)	38 (28.6%)			
Education	32 (11.5%)	32 (24.0%)			
Arts	36 (12.9%)	36 (27.1%)			
Engineering	27 (9.7%)	27 (20.3%)			
Commerce	35 (12.5%)		35 (24.0%)		
Rights	40 (14.3%)		40 (27.4%)		
Computer and information	29 (10.4%)		29 (19.9%)		
Specific education	42 (15.1%)		42 (28.7%)		
Anthropometric measurements					
Weight	77.20 ± 10.80	76.79 ± 10.50	77.58 ± 11.08	−0.612	0.541
Height	168.04 ± 9.19	168.22 ± 8.51	167.88 ± 9.80	0.309	0.757
BMI	27.33 ± 3.21	27.11 ± 3.01	27.54 ± 3.37	−1.104	0.271
Experience and history					
Family history of overweight				1.312	0.277
Yes	73 (26.2%)	39 (29.3%)	34 (23.3%)		
No	206 (73.8%)	94 (70.7%)	112 (76.7%)		
Experience of behavioral therapy				1.408	0.495
Yes	48 (17.2%)	21 (15.8%)	27 (18.5%)		
No	172 (61.6%)	80 (60.2%)	92 (63.0%)		
Do not know	59 (21.1%)	32 (24.1%)	27 (18.5%)		
Experience of calculated calories				1.485	0.476
Yes	90 (32.3%)	43 (32.3%)	47 (32.2%)		
No	140 (50.2%)	63 (47.4%)	77 (52.7%)		
Do not know	49 (17.6%)	27 (20.3%)	22 (15.1%)		
Experience of physical exercises				6.438	0.012
Yes	127 (45.5%)	50 (37.6%)	77 (52.7%)		
No	152 (54.5%)	83 (62.4%)	69 (47.3%)		
Experience of medical therapy				0.432	0.593
Yes	36 (12.9%)	19 (14.3%)	17 (11.6%)		
No	243 (87.1%)	114 (85.7%)	129 (88.4%)		
Motivator/desire to control weight				1.989	0.370
Healthy	32 (11.5%)	17 (12.8%)	15	10.3%	
Appearance	18 (6.5%)	11 (8.3%)	7	4.8%	
Appearance and health	229 (82.1%)	105 (78.9%)	124	84.9%	
HBM constructs					
Perceived severity	3.35 ± 1.08	3.39 ± 1.11	3.32 ± 1.06	0.534	0.594
Emotional/mental health	3.22 ± 1.34	3.21 ± 1.39	3.23 ± 1.30	−0.138	0.890
Physical health/fitness	3.65 ± 1.24	3.69 ± 1.28	3.61 ± 1.21	0.550	0.583
Social professional	3.11 ± 1.20	3.18 ± 1.24	3.04 ± 1.15	1.010	0.313
Perceived susceptibility	3.55 ± 0.93	3.59 ± 0.92	3.50 ± 0.94	0.770	0.442
Lifestyle	3.64 ± 1.03	3.71 ± 0.99	3.58 ± 1.05	1.049	0.295
Environmental	3.30 ± 1.27	3.29 ± 1.27	3.31 ± 1.28	−0.143	0.887
Perceived barriers	3.13 ± 1.32	3.10 ± 1.38	3.15 ± 1.27	−0.310	0.757
Practical concerns	3.17 ± 1.43	3.11 ± 1.49	3.22 ± 1.37	−0.651	0.516
Emotional/mental health	3.23 ± 1.43	3.22 ± 1.48	3.25 ± 1.40	−0.214	0.831
Awareness	2.99 ± 1.43	2.99 ± 1.47	3.00 ± 1.39	−0.071	0.943
Perceived benefits	3.78 ± 0.95	3.71 ± 1.00	3.84 ± 0.90	−1.076	0.283
Emotional/mental health	3.76 ± 1.07	3.64 ± 1.12	3.87 ± 1.00	−1.808	0.072
Physical health/fitness	3.77 ± 1.04	3.74 ± 1.06	3.81 ± 1.02	−0.587	0.557
Social/professional	3.81 ± 1.11	3.77 ± 1.15	3.85 ± 1.09	−0.585	0.559
Cues to action	3.61 ± 0.95	3.61 ± 0.98	3.61 ± 0.93	−0.030	0.976
Internal cues	3.57 ± 1.05	3.53 ± 1.10	3.61 ± 1.00	−0.687	0.493
External cues	3.67 ± 0.98	3.69 ± 0.99	3.66 ± 0.97	0.261	0.794
Perceived self-efficacy	3.57 ± 0.92	3.62 ± 0.94	3.53 ± 0.90	0.838	0.403
Perceived self-efficacy in dieting	3.53 ± 0.96	3.56 ± 1.01	3.51 ± 0.92	0.421	0.674
Habits, preferences	3.55 ± 0.96	3.57 ± 1.00	3.53 ± 0.92	0.393	0.695
Emotional/mental health	3.49 ± 1.09	3.52 ± 1.14	3.46 ± 1.04	0.441	0.660
Perceived self-efficacy in exercise	3.67 ± 0.99	3.77 ± 0.96	3.57 ± 1.01	1.737	0.084
Behavioral intention of weight management	3.18 ± 1.15	3.27 ± 1.15	3.10 ± 1.14	1.272	0.204
Dieting	3.17 ± 1.17	3.26 ± 1.17	3.08 ± 1.17	1.272	0.205
Exercising	3.20 ± 1.31	3.29 ± 1.29	3.12 ± 1.33	1.080	0.281

### 3.1 Baseline characteristics

Except for the experience of physical exercise (*p* = 0.012), there were no statistically significant differences between the IG and CG at T0 regarding demographic characteristics, anthropometric measurements, and the mean score of HBM constructs (Table 2).

### 3.2 Body mass index

**Figure 3:**
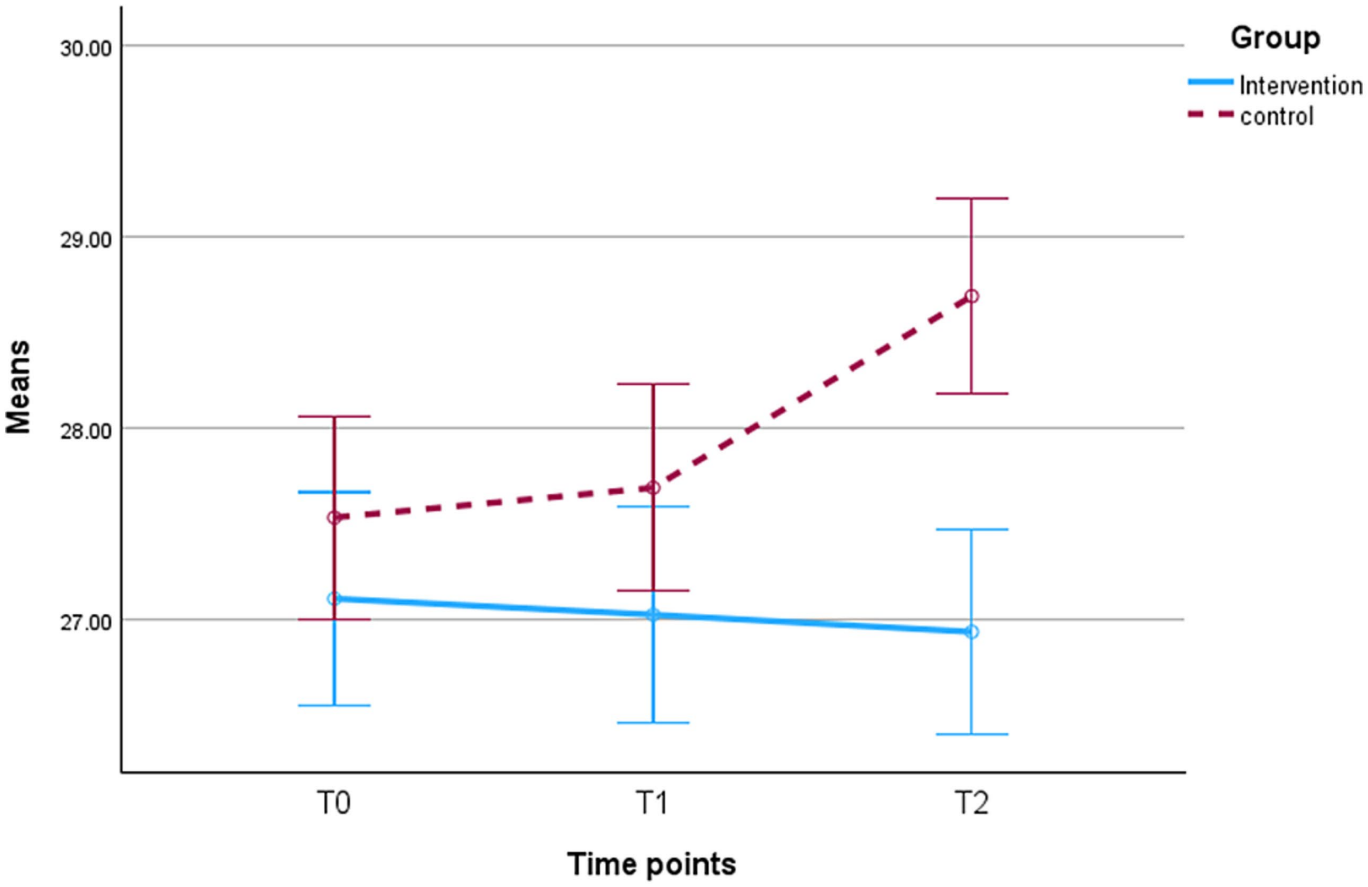
Mean scores of BMI with SE bars (95% CI and ±2 SE); T0, baseline; T1, 2 months; T2, 5 months. Intervention group (*n* = 133); control group (*n* = 146).

It is observed that the mean scores of BMI decreased in the IG compared to the CG at both T1 and T2, as displayed in Figure 3. The independent *t*-test shows no statistically significant difference in BMI between groups at T1 (*p* = 0.091) (Table 3). The statistically significant reduction in BMI was demonstrated in the IG compared to the CG at T2 (−1.75, CI: −2.48, −1.02, *p* < 0.001) and moderate effect size (Cohen’s *d* = −0.568) (Table 3). Also, two-way repeated measures ANOVA reveals a statistically significant small effect between groups: *F*(1, 277) = 6.45, *p* = 0.012, partial η^2^ = 0.023. Interaction effect (Group × Time) is statistically significant: *F*(1.236, 342.358) = 49.72, *p* = <0. 001, partial η^2^ = 0.152. Within-group differences are also statistically significant: *F*(1.236, 342.358) = 29.73, *p* = <0. 001, partial η^2^ = 0.097 (Table 4).

**Table 3:** Mean differences of BMI and HBM constructs between IG (*n* = 133) and CG (*n* = 146) at different time points.

Outcomes	Time points	T value	Mean difference	95% CI	*P-*value	Cohen’s *d* (95% CI)
BMI	T0	−1.10	−0.42	−1.18 to 0.33	0.271	
	T1	−1.70	−0.66	−1.43 to 0.11	0.091	
	T2	−4.73	−1.75	−2.48 to −1.02	<0.001	−0.568
HBM constructs						
Perceived severity	T0	0.53	0.07	−0.18 to 0.32	0.594	
	T1	0.99	0.12	−0.12 to 0.35	0.324	
	T2	1.63	0.16	−0.03 to 0.35	0.105	
Emotional/mental health	T0	−0.14	−0.02	−0.34 to 0.29	0.890	
	T1	0.35	0.05	−0.24 to 0.34	0.729	
	T2	1.46	0.17	−0.06 to 0.4	0.146	
Physical health/fitness	T0	0.55	0.08	−0.21 to 0.38	0.583	
	T1	0.45	0.06	−0.2 to 0.33	0.653	
	T2	0.94	0.11	−0.12 to 0.34	0.350	
Social professional	T0	1.01	0.14	−0.13 to 0.43	0.313	
	T1	1.87	0.25	−0.01 to 0.52	0.063	
	T2	1.79	0.21	−0.02 to 0.44	0.075	
Perceived susceptibility	T0	0.77	0.08	−0.13 to 0.3	0.442	
	T1	2.59	0.26	0.06–0.46	0.010	0.310
	T2	3.95	0.40	0.2–0.6	<0.001	0.474
Lifestyle	T0	1.05	0.13	−0.11 to 0.37	0.295	
	T1	2.48	0.27	0.05–0.48	0.014	0.297
	T2	4.49	0.46	0.26–0.67	<0.001	0.539
Environmental	T0	−0.14	−0.02	−0.32 to 0.28	0.887	
	T1	1.64	0.25	−0.05 to 0.55	0.102	
	T2	1.59	0.23	−0.05 to 0.52	0.112	
Perceived barriers	T0	−0.31	−0.05	−0.36 to 0.26	0.757	
	T1	−1.30	−0.19	−0.49 to 0.09	0.195	
	T2	−3.19	−0.45	−0.73 to −0.17	0.002	−0.382
Practical concerns	T0	−0.65	−0.11	−0.45 to 0.22	0.516	
	T1	−1.98	−0.33	−0.65 to −0.01	0.049	−0.237
	T2	−4.43	−0.70	−1.01 to −0.39	<0.001	−0.531
Emotional/mental health	T0	−0.21	−0.04	−0.38 to 0.3	0.831	
	T1	−1.23	−0.20	−0.52 to 0.12	0.221	
	T2	−3.30	−0.52	−0.84 to −0.21	0.001	−0.395
Awareness	T0	−0.07	−0.01	−0.35 to 0.32	0.943	
	T1	−0.51	−0.08	−0.39 to 0.23	0.613	
	T2	−1.23	−0.19	−0.49 to 0.11	0.218	
Perceived benefits	T0	−1.08	−0.12	−0.35 to 0.1	0.283	
	T1	2.97	0.34	0.11–0.56	0.003	0.357
	T2	4.61	0.48	0.28–0.69	<0.001	0.553
Emotional/mental health	T0	−1.81	−0.23	−0.48 to 0.02	0.072	
	T1	2.62	0.31	0.08–0.54	0.009	0.314
	T2	5.16	0.57	0.35–0.79	<0.001	0.618
Physical health/fitness	T0	−0.59	−0.07	−0.32 to 0.17	0.557	
	T1	2.78	0.35	0.1–0.6	0.006	0.333
	T2	3.88	0.45	0.23–0.68	<0.001	0.465
Social/professional	T0	−0.58	−0.08	−0.34 to 0.18	0.559	
	T1	2.75	0.35	0.1–0.6	0.006	0.330
	T2	3.35	0.40	0.17–0.64	<0.001	0.401
Cues to action	T0	−0.03	−0.03	−0.23 to 0.22	0.976	
	T1	2.17	0.24	0.02–0.45	0.030	0.261
	T2	3.77	0.40	0.19–0.6	<0.001	0.452
Internal cues	T0	−0.69	−0.09	−0.33 to 0.16	0.493	
	T1	2.32	0.28	0.04–0.52	0.021	0.278
	T2	5.24	0.64	0.4–0.88	<0.001	0.628
External cues	T0	0.26	0.03	−0.2 to 0.26	0.794	
	T1	1.58	0.19	−0.05 to 0.43	0.116	
	T2	1.27	0.16	−0.08 to 0.4	0.102	
Perceived self-efficacy	T0	0.84	0.09	−0.12 to 0.31	0.403	
	T1	2.40	0.26	0.05–0.47	0.017	0.288
	T2	3.83	0.41	0.2–0.62	<0.001	0.459
Perceived self-efficacy in dieting	T0	0.42	0.05	−0.18 to 0.28	0.674	
	T1	2.33	0.26	0.04–0.48	0.021	0.279
	T2	3.41	0.4	0.17–0.63	<0.001	0.409
Habits, preferences	T0	0.39	0.04	−0.18 − 0.27	0.695	
	T1	2.10	0.24	0.01–0.47	0.037	0.251
	T2	3.29	0.4	0.16–0.64	0.001	0.394
Emotional/mental health	T0	0.44	0.06	−0.2 to 0.31	0.660	
	T1	2.48	0.31	0.06–0.56	0.014	0.297
	T2	3.08	0.4	0.14–0.66	0.002	0.370
Perceived self-efficacy in exercise	T0	1.74	0.2	−0.03 to 0.44	0.084	
	T1	2.40	0.26	0.05–0.47	0.017	0.288
	T2	3.38	0.43	0.18–0.69	0.001	0.405
Behavioral intention of weight management	T0	1.27	0.17	−0.09 to 0.44	0.204	
	T1	3.22	0.48	0.19–0.77	0.001	0.387
	T2	4.90	0.71	0.43–1	<0.001	0.588
Dieting	T0	1.27	0.18	−0.09 to 0.45	0.205	
	T1	3.27	0.49	0.2–0.79	0.001	0.362
	T2	4.85	0.73	0.43–1.02	<0.001	0.582
Exercising	T0	1.08	0.17	−0.14 to 0.48	0.281	
	T1	2.81	0.45	0.13–0.77	0.005	0.337
	T2	4.38	0.69	0.38–1	<0.001	0.525

**Table 4:** Results of two-way repeated measures analysis of variance of BMI and HBM constructs at different time points (IG; *n* = 133) and (CG; *n* = 146).

Outcomes	Time points	IG (*n* = 133)	CG (*n* = 146)	Two-way repeated measures ANOVA								
				Interaction group × time			Within-groups			Between-groups		
		Mean ± SD	Mean ± SD	*F*-value	*P*-value	partial η^2^	*F*-value	*P*-value	partial η^2^	*F*-value	*P*-value	partial η^2^
BMI	T0	27.11 ± 3.01	27.54 ± 3.37	49.72	<0.001	0.152	29.73	<0.001	0.097	6.45	0.012	0.023
	T1	27.03 ± 3.03	27.69 ± 3.45									
	T2	26.94 ± 2.61	28.69 ± 3.47									
HBM constructs												
Perceived severity	T0	3.39 ± 1.11	3.32 ± 1.06	0.762	0.439	0.003	28.45	<0.001	0.093	1.13	0.289	0.004
	T1	3.59 ± 1.04	3.47 ± 0.94									
	T2	3.70 ± 0.83	3.54 ± 0.80									
Emotional/mental health	T0	3.21 ± 1.39	3.23 ± 1.30	2.786	0.072	0.01	46.25	<0.001	0.143	0.24	0.623	0.001
	T1	3.46 ± 1.30	3.41 ± 1.17									
	T2	3.70 ± 0.98	3.53 ± 0.97									
Physical health/fitness	T0	3.69 ± 1.28	3.61 ± 1.21	0.185	0.782	0.001	8.29	0.001	0.029	0.44	0.506	0.002
	T1	3.81 ± 1.18	3.75 ± 1.08									
	T2	3.85 ± 0.97	3.75 ± 0.97									
Social professional	T0	3.18 ± 1.24	3.04 ± 1.15	0.849	0.404	0.003	25.22	<0.001	0.083	2.71	0.101	0.01
	T1	3.45 ± 1.19	3.20 ± 1.08									
	T2	3.50 ± 0.99	3.29 ± 0.96									
Perceived susceptibility	T0	3.59 ± 0.92	3.50 ± 0.94	8.003	0.001	0.028	26.55	<0.001	0.087	6.93	0.009	0.024
	T1	3.85 ± 0.82	3.59 ± 0.87									
	T2	4.03 ± 0.68	3.63 ± 0.96									
Lifestyle	T0	3.71 ± 0.99	3.58 ± 1.05	8.550	0.001	0.03	18.03	<0.001	0.061	8.00	0.005	0.028
	T1	3.93 ± 0.81	3.66 ± 0.98									
	T2	4.12 ± 0.62	3.66 ± 1.03									
Environmental	T0	3.29 ± 1.27	3.31 ± 1.28	2.531	0.097	0.009	16.31	<0.001	0.056	1.43	0.234	0.005
	T1	3.66 ± 1.33	3.41 ± 1.22									
	T2	3.80 ± 1.27	3.57 ± 1.16									
Perceived barriers	T0	3.10 ± 1.38	3.15 ± 1.27	36.57	<0.001	0.117	1.24	0.280	0.004	2.47	0.117	0.009
	T1	2.99 ± 1.30	3.19 ± 1.19									
	T2	2.89 ± 1.33	3.34 ± 1.05									
Practical concerns	T0	3.11 ± 1.49	3.22 ± 1.37	33.06	<0.001	0.107	7.53	0.002	0.026	5.64	0.018	0.02
	T1	3.02 ± 1.44	3.35 ± 1.32									
	T2	2.95 ± 1.50	3.65 ± 1.12									
Emotional/mental health	T0	3.22 ± 1.48	3.25 ± 1.40	26.34	<0.001	0.087	1.64	0.202	0.006	2.52	0.114	0.009
	T1	3.13 ± 1.42	3.33 ± 1.31									
	T2	3.02 ± 1.48	3.55 ± 1.17									
Awareness	T0	2.99 ± 1.47	3.00 ± 1.39	9.78	<0.001	0.034	46.21	<0.001	0.143	0.35	0.555	0.001
	T1	2.84 ± 1.32	2.92 ± 1.32									
	T2	2.70 ± 1.26	2.89 ± 1.31									
Perceived benefits	T0	3.71 ± 1.00	3.84 ± 0.90	25.46	<0.001	0.084	28.01	<0.001	0.092	5.618	0.018	0.02
	T1	4.14 ± 0.86	3.80 ± 1.01									
	T2	4.35 ± 0.75	3.86 ± 0.97									
Emotional/mental health	T0	3.64 ± 1.12	3.87 ± 1.00	31.07	<0.001	0.101	16.91	<0.001	0.058	4.442	0.036	0.016
	T1	4.18 ± 0.87	3.88 ± 1.07									
	T2	4.29 ± 0.72	3.72 ± 1.10									
Physical health/fitness	T0	3.74 ± 1.06	3.81 ± 1.02	17.39	<0.001	0.059	28.77	<0.001	0.094	4.922	0.027	0.017
	T1	4.11 ± 0.97	3.76 ± 1.12									
	T2	4.36 ± 0.87	3.90 ± 1.06									
Social/professional	T0	3.77 ± 1.15	3.85 ± 1.09	13.22	<0.001	0.046	28.37	<0.001	0.093	4.012	0.046	0.014
	T1	4.14 ± 0.95	3.79 ± 1.16									
	T2	4.40 ± 0.88	4.00 ± 1.12									
Cues to action	T0	3.61 ± 0.98	3.61 ± 0.93	29.70	<0.001	0.097	23.80	<0.001	0.079	3.995	0.047	0.014
	T1	3.86 ± 0.86	3.63 ± 0.95									
	T2	3.98 ± 0.73	3.58 ± 0.99									
Internal cues	T0	3.53 ± 1.10	3.61 ± 1.00	34.55	<0.001	0.111	36.98	<0.001	0.118	6.106	0.014	0.022
	T1	3.99 ± 1.02	3.71 ± 1.01									
	T2	4.24 ± 0.83	3.61 ± 1.16									
External cues	T0	3.69 ± 0.99	3.66 ± 0.97	5.30	0.013	0.019	1.30	0.266	0.005	1.16	0.281	0.004
	T1	3.74 ± 0.96	3.55 ± 1.06									
	T2	3.71 ± 0.96	3.56 ± 1.08									
Perceived self-efficacy	T0	3.62 ± 0.94	3.53 ± 0.90	24.05	<0.001	0.08	14.13	<0.001	0.049	5.825	0.016	0.021
	T1	3.65 ± 0.92	3.39 ± 0.89									
	T2	3.85 ± 0.89	3.44 ± 0.90									
Perceived self-efficacy in dieting	T0	3.56 ± 1.01	3.51 ± 0.92	18.12	<0.001	0.061	15.44	<0.001	0.053	4.635	0.032	0.016
	T1	3.62 ± 0.98	3.36 ± 0.89									
	T2	3.85 ± 1.02	3.45 ± 0.94									
Habits, preferences	T0	3.57 ± 1.00	3.53 ± 0.92	14.42	<0.001	0.049	15.39	<0.001	0.053	4.247	0.040	0.015
	T1	3.61 ± 1.01	3.37 ± 0.92									
	T2	3.87 ± 1.05	3.47 ± 0.97									
Emotional/mental health	T0	3.52 ± 1.14	3.46 ± 1.04	17.46	<0.001	0.059	7.99	0.001	0.028	4.30	0.039	0.015
	T1	3.66 ± 1.11	3.35 ± 1.01									
	T2	3.81 ± 1.15	3.40 ± 1.03									
Perceived self-efficacy in exercise	T0	3.77 ± 0.96	3.57 ± 1.01	5.97	0.004	0.021	9.60	<0.001	0.034	7.23	0.008	0.025
	T1	3.65 ± 0.92	3.39 ± 0.89									
	T2	3.84 ± 1.10	3.41 ± 1.05									
Behavioral intention of weight management	T0	3.27 ± 1.15	3.10 ± 1.14	20.06	<0.001	0.068	0.826	0.407	0.003	11.35	<0.001	0.039
	T1	3.43 ± 1.31	2.95 ± 1.17									
	T2	3.59 ± 1.27	2.88 ± 1.15									
Dieting	T0	3.26 ± 1.17	3.08 ± 1.17	18.834	<0.001	0.064	1.414	0.244	0.005	11.468	<0.001	0.04
	T1	3.41 ± 1.34	2.91 ± 1.19									
	T2	3.59 ± 1.32	2.87 ± 1.17									
Exercising	T0	3.29 ± 1.29	3.12 ± 1.33	15.652	<0.001	0.053	0.240	0.725	0.001	8.618	0.004	0.03
	T1	3.45 ± 1.36	3.00 ± 1.32									
	T2	3.59 ± 1.36	2.89 ± 1.28									

ANCOVA models adjusting for sex and age were performed, and revealed that the variables were not statistically significant confounders. Additionally, no statistically significant sex-by-intervention or age-by-intervention interactions were observed.

### 3.3 Perceived severity

**Figure 4:**
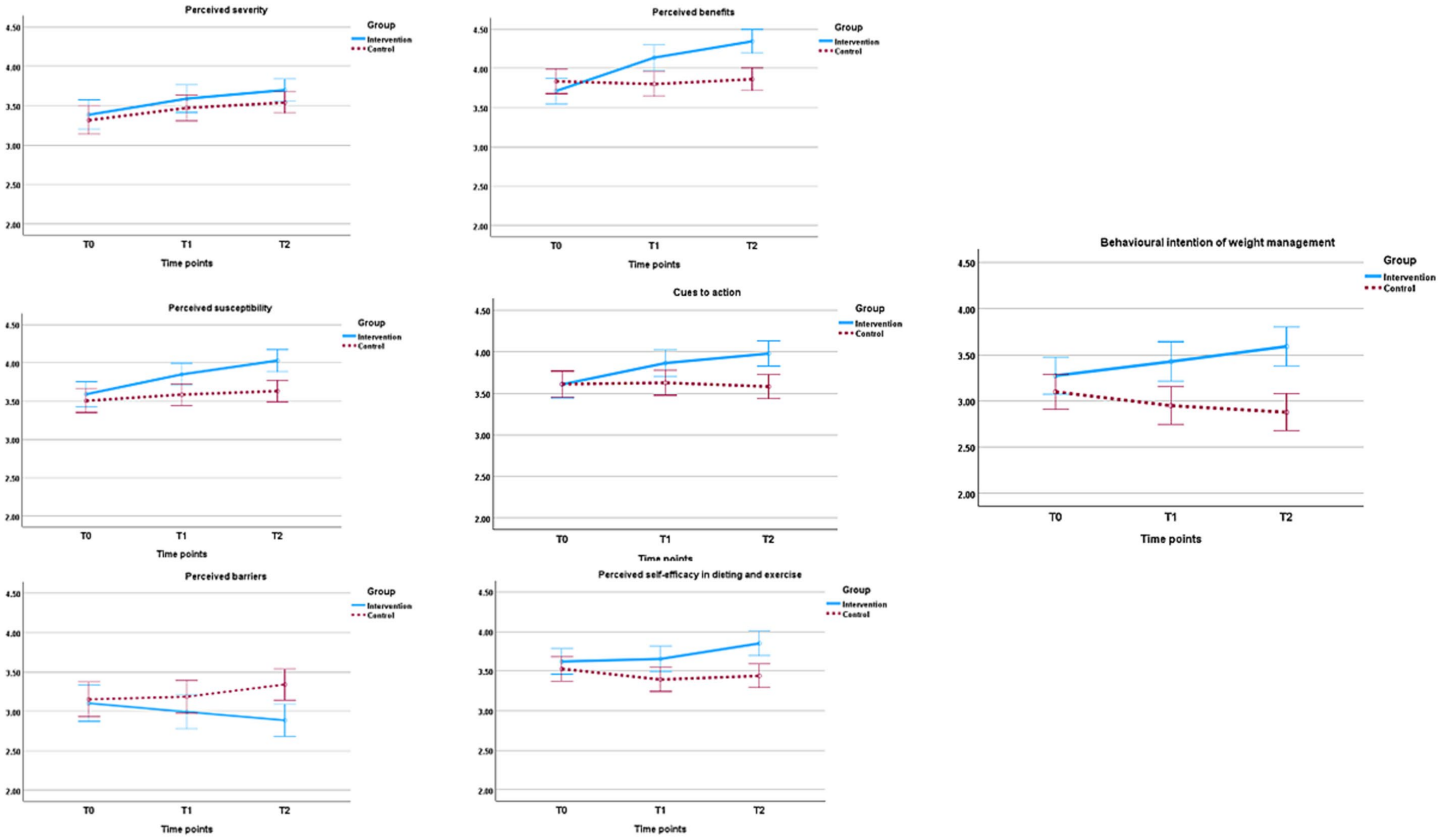
Mean scores of HBM constructs with SE bars (95% CI and ±2 SE); T0, baseline; T1, 2 months; T2, 5 months. Intervention group (*n* = 133); control group (*n* = 146).

Figure 4 shows that perceived severity increases in both groups at different time points. This progress in the two groups did not differ statistically between groups at T1 (*p* = 0.324) or T2 (*p* = 0.105) as revealed in Table 3 for the perceived severity and subscales. However, there is a statistically significant difference within-group: *F*(1.589, 440.089) = 28.45, p = <. 001, partial η^2^ = 0.093. Similar results were revealed in the subscales of perceived severity (emotional/mental health, physical health/fitness, and social professional) [*F*(1.69, 469.35) = 46.25, *p* = <0.001, partial η^2^ = 0.0143], [*F*(1.60, 442.40) = 8.29, *p* = 0.001, partial η^2^ = 0.029], and [*F*(1.57, 435.83) = 25.22, *p* = <0.001, partial η^2^ = 0.083] respectively (Table 4).

### 3.4 Perceived susceptibility

Conversely, Figure 3 shows increased perceived susceptibility in the IG versus the CG. The intervention has a statistically significant positive effect on the adolescents in the intervention group to perceive their susceptibility to overweight consequences, compared to the adolescents in the control group at T1 (0.26, CI: 0.06–0.46, *p* = 0.010) and T2 (0.40, CI: 0.2–0.6, *p* < 0.001) (Table 3). Furthermore, there are statistical differences within-group over time and a statistically significant interaction effect (Group × Time) [*F*(1.68, 466.47) = 26.55, *p* < 0.001, partial η^2^ = 0.087], and [*F*(1.68, 466.47) = 8.00, *p* = 0.001, partial η^2^ = 0.028] respectively (Table 4). What is noted, the statistically significant progress in the perceived susceptibility in IG is attributed to statistically significant change in lifestyle subscale at T1 (0.27, CI: 0.05–0.48, *p* = 0.014) and T2 (0.46, CI: 0.26–0.67, *p* < 0.001) compared to CG, and the statistically significant main effect within groups overtime [*F*(1.57, 435.81) = 18.03, *p* < 0.001, partial η^2^ = 0.061] (Table 4). The environmental subscale did not differ statistically between groups at different time points (all *p* > 0.05) (Table 4).

### 3.5 Perceived barriers

The intervention group (IG) experiences a reduction in perceived barriers and subscales compared to the control group (CG), as shown in Figure 4; Table 3. Still, this difference is not statistically significant at T1 (all *p* > 0.05) (Table 3). However, the difference becomes statistically significant at T2 (−0.45, CI: −0.73 to −0.17, *p* = 0.002), though with a small effect size (Cohen’s *d* = −0.382) (Table 3). In addition, it is observed that there are no statistically significant changes within groups over time for the total perceived barriers (Table 4). The statistically significant difference of total perceived barriers between IG and CG at T2 may be attributed to the statistically significant interaction effect (Group × Time) for the total perceived barriers: *F*(1.37, 379.48) = 36.57, *p* < 0.001, partial η^2^ = 0.117 and subscales; practical concerns: *F*(1.44,399.82) = 33.06, *p* < 0.001, partial η^2^ = 0.107, emotional mental health: *F*(1.229, 340.37) = 26.34, *p* < 0.001, partial η^2^ = 0.087, awareness: *F*(1.435, 397.46) = 9.78, *p* < 0.001, partial η^2^ = 0.034.

### 3.6 Perceived benefits

Perceived benefits increased in the IG, compared to the CG, as shown in Figure 4. There are statistically significant differences between groups at T1 and T2 regarding total perceived benefits and subscales (all *p* < 0.05) (Table 3). In addition, there are statistically significant effects of Interaction (Group × Time) and within-group (all *p* < 0.05) (Table 4).

### 3.7 Perceived cues to action

Concerning the differences in perceived internal and external cues to action between the IG and CG. Specifically, internal cues were statistically significantly different between the IG and CG at both T1 (0.28, CI: 0.04–0.52, *p* = 0.021) and T2 (0.64, CI: 0.40–0.88, *p* < 0.001) (Table 3). External cues, however, were not statistically different between the two groups at T1 (*p* = 0.116) or T2 (*p* = 0.102) (Table 3), and neither did they change statistically within-groups over time (*p* = 0.266) (Table 4). The overall total change in cues to action was statistically significant between groups at T1 (0.24, CI: 0.02–0.45, *p* = 0.03) and T2 (0.40, CI: 0.19–0.60, *p* < 0.001) (Table 3), within groups: *F*(1.69, 467.69) = 23.80, *p* < 0.001, partial η^2^ = 0.079, and interaction: *F*(1.69, 467.69) = 3.99, *p* < 0.047, partial η^2^ = 0.014 (Table 4). This change in the total cues to action is mainly related to the statistically significant improvement in internal cues.

### 3.8 Perceived efficacy for dieting and exercise

Table 3 presents the participants in the intervention group (IG) showed a statistically significant increase in total perceived efficacy for dieting and exercise, compared to the control group (CG) at T1 (0.26, CI:0.05–0.47, *p* = 0.017) and T2 (0.41, CI:0.20–0.62, *p* < 0.00). Also, it is revealed statistically significant effect within-groups: *F*(1.42, 393.29) = 14.13, *p* < 0.001, partial η^2^ = 0.049, and statistically significant interaction effect between group and time: *F*(1.42, 393.29) = 24.05, *p* < 0.001, partial η^2^ = 0.08. At the same time, there are statistically significant changes for all subscales of perceived efficacy (all *p* < 0.0.05).

### 3.9 Perceived behavioral intention of weight management in dieting and exercise

The means scores of behavioral intention of weight management decreased from T0 to T2, while the means increased obviously in the IG over timepoints (Figure 4). Compared to the CG, the participants in the IG perceived statistically significant behavioral intention in dieting and exercise at T1 (0.48, CI: 0.19–0.77, *p* = 0.001) and T2 (0.71, CI: 0.43–1.00, *p* < 0.001) (Table 3). However, these improvements did not demonstrate a statistically significant effect within groups over time (*p* = 0.407) (Table 4). This indicates a positive impact on behavioral intention, but this impact was not sustained among participants over time.

## 4 Discussion

This RCT investigated the effectiveness of a tailored multichannel intervention in promoting weight loss and enhancing health beliefs among overweight adolescents, informed by HBM. The findings demonstrate that the intervention had a statistically significant impact on BMI reduction and several HBM constructs, validating the hypothesis that tailored multichannel interventions can effectively influence weight loss and management behaviors among adolescents. In line with the current trial, previous research and systematic reviews have reported significant decreases in BMI when interventions are grounded in theory, tailored to individuals, and provided through various channels over a sufficient period (19).

### 4.1 BMI

The primary outcome—BMI—decreased significantly in the IG compared to the CG by the end of the intervention at 5 months, with a moderate effect size. This finding aligns with previous research that proves sustained tailored interventions targeting specific cognitive and behavioral determinants can produce clinically meaningful changes in body weight (16). The statistically significant interaction effect between group and time further supports the assertion that sustained, multichannel engagement over time contributes to positive outcomes. In contrast, the lack of a statistically significant difference between groups at 2 months suggests that early intervention phases affect awareness and motivation, rather than resulting in immediate weight changes (63). This interpretation is consistent with previous studies. For example, the trial aimed to determine the effectiveness of 3 months of nutritional education based on the HBM on self-esteem and BMI of overweight adolescent girls. The study revealed no significant difference in mean BMI score between the IG and CG. There was no statistically significant difference between groups at 2 months [IG (26.82 ± 1.42), CG (27.19 ± 1.55), *p* = 0.170] or at 3 months from the intervention [IG (26.70 ± 1.38), CG (27.13 ± 1.56), *p* = 0.090] (64). Similarly, the RCT, which evaluated the effectiveness of a 10-week theory-based intervention to manage overweight among adolescents, demonstrated a reduction in BMI among overweight adolescents. Mean scores of BMI of the participants in the IG were reduced from 28.10 to 27.93 kg/m^2^ (*p* = 0.001) from baseline to immediate post-intervention and further decreased to 27.80 kg/m^2^ (*p* < 0.001) from baseline to 3-month follow-up. However, there were no statistically significant differences between groups, neither post-intervention (*p* = 0.175) nor follow-up (*p* = 0.093) (65). These findings suggest that short-term intervention may not be sufficient to achieve statistically significant outcomes. Supporting this, a systematic review assessing weight loss trajectories in a 12-month randomized crossover study found that participants experienced statistically significant weight loss between three and 6 months of intervention (66).

### 4.2 HBM

The intervention effectively improved several key HBM constructs. Similar to the current results, a trial was conducted to assess the impact of educational intervention, based on HBM and collaborative learning techniques, on diet quality in adolescents. The study elaborated that there were statistically significant improvements regarding all subscales of HBM (perceived severity, perceived susceptibility, perceived barriers, perceived benefits, perceived self-efficacy, cues to action, and behavioral intention of weight management). HBM’s constructs were significantly improved in the IG, and mean differences were increased after the intervention (*p* < 0.001), which was statistically significantly different from the CG (*p* = 0.001) (67). Another trial was conducted to investigate the efficacy of a training program for overweight women based on the HBM and social support approach. It was reported “After the intervention, a statistically significant difference was observed between the two groups on perceived benefits [*t* (71) = 3.55, *p* < 0.001], perceived barriers [*t* (71) = 3.60, *p* = 0.001], cue to action [*t* (70) = 1.91, *p* = 0.03], and self-efficacy [*t* (71) = 3.45, *p* < 0.001] with very large to small effect sizes.” In addition, the participants within the IG experienced statistically significant improvements. “Post-test scores on perceived benefits [*t*(33) = 2.82, *p* = 0.02], cue to action [*t*(33) = 2.17, *p* = 0.03], and self-efficacy [*t*(33) = 2.46, *p* = 0.01] differed significantly from pre-test scores with medium effect sizes” (68).

The results of the present study are consistent with those of Rabiei et al., who assessed the effectiveness of 3 months of nutritional education based on the HBM on self-esteem and BMI in overweight adolescent girls. They discovered that the intervention group’s mean score in knowledge, perceived susceptibility, perceived severity, perceived benefits, and, finally, self-esteem before and after the intervention differed significantly among adolescents in the IG from the CG (all *p* < 0.001) (64). This finding is congruent with the results reported by Salem and Said, who investigated the effect of HBM-based nutrition education on the dietary habits of adolescent girls. It was found that nutrition education changes adolescents’ views regarding healthy eating habits. It was revealed that the perceived susceptibility, severity, benefits, barriers, self-efficacy, and cues to action were statistically significantly improved after intervention (*p* < 0.001) (69).

Perceived severity increased significantly in both groups. It indicates a growing awareness of the risks of being overweight. This statistically significant progress within groups is likely due to the general education provided to both groups. Perceived susceptibility significantly increased in the IG at different timepoints, especially in the lifestyle subscale, suggesting that adolescents in the IG became more aware of their risk of overweight-related health issues. This is critical as higher perceived susceptibility has been linked to increased motivation for health behavior change (70). Perceived benefits, internal cues to action, and self-efficacy in dieting and exercise significantly improved in the IG compared to the CG. The observed improvements are in agreement with prior HBM-based interventions targeting adolescent weight management (68). These findings reinforce that altering health beliefs is central and essential for promoting long-term adherence to health-promoting behaviors. The increase in internal cues (e.g., emotional triggers or self-awareness) suggests that adolescents became more attuned to their internal motivators for change, a critical factor for sustainable weight management (71). Saghafi-Asl et al. highlighted that cues to action and perceived self-efficacy were significantly associated with weight loss behavioral intention (10). The multichannel delivery method employed in this study may account for the more significant enhancements in cognitive and motivational constructs when compared to single-channel or solely educational interventions documented in other research (19). Consistent interaction through in-person meetings, telephone counseling, and WhatsApp communications likely bolstered learning, improved self-monitoring, and offered repeated internal prompts to take action (16).

Conversely, certain findings differ from those of comparable studies. External cues to action showed no statistically significant changes between groups or within groups over time. Furthermore, the perceived barriers construct demonstrated a statistically significant reduction only at the end of the intervention in the IG, indicating a delayed effect of the intervention on overcoming obstacles to behavior change. This contrasts with certain interventions that included family participation or wider community involvement, which have shown more significant decreases in perceived obstacles and more robust external motivational factors (72). Despite the adolescents’ awareness of overweight-related health consequences, overweight management is the main challenge facing this vulnerable group (73). Several lifestyle interventions were provided without revealing positive outcomes (74). There were various barriers to implementation. Barriers include personal factors, the unavailability of healthy foods, and a lack of community support. Furthermore, the healthcare system is designed to focus on disease management instead of promoting health (73).

The review, which aimed to summarize the evidence on the effectiveness of interventions for adolescent overweight in limited socioeconomic resource settings, concluded that no definite intervention approaches were particularly successful in managing overweight among disadvantaged adolescents (74). However, the evidence suggests that integrating community support in intervention delivery seems to be a useful strategy (72). Engagement of educational institutions or family could enhance external motivational sources (75). The educational institution is a familiar environment for enhancing healthy lifestyles (76). Furthermore, it was suggested that school programs include weight management strategies (65).

Finally, behavioral intention for weight management in dieting and exercise significantly improved in the IG at both T1 and T2 compared to the CG. However, this improvement was not sustained within the groups over time. This indicates that the intervention initially increases intentions to change, but these intentions may not have been maintained. Highlighting the need for continued reinforcement or follow-up beyond the intervention period.

## 5 Limitations

The results of this RCT are consistent with current evidence indicating that tailored, multichannel lifestyle interventions can successfully encourage weight loss and foster positive behavioral changes in adolescents. However, several limitations should be noted. First, BMI, while widely used, does not account for body composition variability such as muscle mass or fat distribution. Also, although guidelines support a BMI cutoff (BMI ≥ 25 kg/m^2^) for late adolescents, BMI-for-age sensitivity analysis may be considered a limitation of the study. Second, a long questionnaire of HBM constructs may present a burden on adolescents. Third, the progress evaluation, intervention adherence, and individual understanding of the intervention were not assessed adequately. Fourth, the college-based setting may limit the generalizability of findings to other age groups or non-academic populations. Fifth, the absence of family participation may have influenced the outcomes, given the importance of familial support in adolescent health behaviors. Sixth, improvements in behavioral intention were not sustained over time, suggesting a need for longer intervention or follow-up periods. Finally, although the statistical analysis was performed by an independent researcher, a bias in estimating the effectiveness of the intervention is anticipated due to the open-label design. Also, the study may be underpowered to detect cluster-level effects. Moreover, LOCF may have methodological limitations. Future research with prolonged follow-up and larger samples may consider multiple-imputation or mixed-effects approaches.

## 6 Recommendations

The results of the study could not be generalized. However, the findings of this research carry significant implications for health policy and clinical practice. Policymakers must consider weight management initiatives within school systems, supported by digital health platforms, to ensure ongoing engagement. Incorporating such evidence-based strategies into national adolescent health policies could reduce obesity-related morbidity and nurture lifelong healthy habits. Furthermore, promoting intersectoral collaboration among healthcare systems, educational entities, and families is vital for maintaining behavioral changes beyond the duration of the intervention. Expanding these initiatives, with the support of community resources and policy measures, would contribute to adolescent wellbeing and public health objectives.

## 7 Conclusion

This research highlights various positive methodological and conceptual aspects that add to the expanding evidence base regarding adolescent weight management. First, it stands out as one of the limited randomized controlled trials that thoroughly assesses a tailored multichannel intervention specifically designed for adolescents and based on the Health Belief Model (HBM). The study provides strong evidence that a tailored multichannel intervention based on the theoretical framework HBM significantly improves BMI and multiple health beliefs related to weight loss among overweight adolescents. Second, the application of a theoretical framework improved the intervention’s ability to address cognitive, affective, and behavioral factors influencing weight-related behaviors. Third, the multichannel strategy—combining in-person sessions, telephone counseling, and WhatsApp follow-ups—promoted ongoing engagement and reinforced behavioral changes through repeated prompts for action. Fourth, the design of the intervention was backed by a multidisciplinary team, which ensured the content’s validity and its relevance to the psychosocial and lifestyle contexts of adolescents. Fifth, the randomization process reduced contamination bias among participants. The inclusion of several assessment points (baseline, mid-intervention, and post-intervention) allowed for a thorough evaluation of changes over time. Moreover, the adherence to CONSORT guidelines further strengthens methodological rigor, internal validity, and reproducibility. In summary, these strengths highlight the methodological robustness of the study.

## Data Availability

The raw data supporting the conclusions of this article will be made available by the authors, without undue reservation. The data were submitted to Dryad platform data repository, doi: 10.5061/dryad.sj3tx96k5.
